# Activation of the Nrf2-ARE Signaling Pathway Mediates the Neuroprotective Effects of Exercise in a Mouse Model of Chronic Parkinson’s Disease

**DOI:** 10.3390/ijms27167439

**Published:** 2026-08-20

**Authors:** Shuwei Zhang, Dong Yang, Changfa Tang, Yu Zhang

**Affiliations:** College of Physical Education, Hunan Normal University, 529 Lu Shan Nan Road, Changsha 410012, China; 18804736223@163.com (S.Z.);

**Keywords:** Parkinson’s disease, treadmill exercise, Nrf2-ARE pathway, dopaminergic neurons, oxidative stress, neuroinflammation

## Abstract

Currently, effective disease-modifying treatments for Parkinson’s disease (PD) remain lacking. Although exercise has been confirmed to exert neuroprotective effects against PD, its specific molecular mechanisms remain unclear. In particular, the causal role of the Nuclear Factor E2-related Factor 2 (Nrf2)–antioxidant response element (ARE) signaling axis in this process has not been clearly elucidated. This study aimed to provide direct causal evidence that regular treadmill exercise protects the substantia nigra pars compacta (SNc)–striatal dopaminergic system by activating the Nrf2-ARE pathway. The results showed that exercise significantly improved motor function deficits in PD mice, preserved the number of TH-positive neurons, and restored striatal dopamine homeostasis. Mechanistically, exercise activated the Nrf2-ARE pathway, thereby inhibiting the accumulation of mitochondrial ROS (mtROS) and the activation of pro-inflammatory amoeboid microglia in the substantia nigra. Crucially, the improvements in behavioral, neuropathological, and neurochemical indicators induced by exercise were completely reversed, returning to levels comparable to those in the sedentary model group. In conclusion, this study provides clear pharmacological evidence that the neuroprotective effect of regular treadmill exercise against chronic PD strictly depends on the Nrf2-ARE pathway. These findings indicate that Nrf2 is a key mechanistic node linking exercise and neuroprotection, providing reliable preclinical evidence for Nrf2-targeted disease-modifying strategies in PD rehabilitation.

## 1. Introduction

Parkinson’s disease (PD) is a neurodegenerative disorder primarily characterized by the progressive loss of dopaminergic neurons in the substantia nigra pars compacta of the midbrain [1,2]. Its complex pathogenesis involves a vicious cycle of interconnected pathological processes, including oxidative stress, mitochondrial dysfunction, and neuroinflammation [3]. Currently, clinical management relies predominantly on dopamine replacement therapies such as levodopa [4,5]. While these treatments alleviate motor symptoms, they cannot stop the underlying disease progression. For this reason, the development of disease-modifying therapies (DMTs) capable of genuinely slowing neurodegeneration has become the most urgent unmet need in PD research.

As part of the cell’s core defense system against oxidative damage, the nuclear factor E2-related factor 2 (Nrf2)–antioxidant response element (ARE) signaling pathway plays a pivotal role [6,7]. Under physiological conditions, Nrf2 is anchored in the cytoplasm by Keap1 and constitutively degraded [8,9]. Upon exposure to oxidative stress, Nrf2 dissociates from Keap1 and translocates into the nucleus, where it binds to ARE sequences to initiate the transcription of cytoprotective target genes such as HO-1 and NQO1 [10]. A large body of evidence confirms that dysregulation of the Nrf2 pathway is closely linked to the pathogenesis of Parkinson’s disease (PD): both PD patients and preclinical PD models show downregulated expression of Nrf2 and its downstream target genes [11]. Conversely, Nrf2 activators such as dimethylfumarate (DMF) produce significant neuroprotective effects in PD models, confirming that Nrf2 is a highly promising therapeutic target for the condition [12].

In addition to pharmacological interventions, physical exercise has emerged as a high-value non-pharmacological intervention for Parkinson’s disease (PD) [13,14,15]. Both clinical and preclinical studies consistently show that exercise improves motor function and reduces damage to dopaminergic neurons, through mechanisms that include increased neurotrophic factor secretion, improved mitochondrial function, and suppression of oxidative stress and neuroinflammation [16,17]. Notably, multiple studies have detected elevated Nrf2 activity in exercise-treated animals, indicating that the protective effects of exercise may be associated with Nrf2 activation [18]. However, current research has largely remained at the stage of observational correlation, leaving a critical unresolved scientific question: is Nrf2 activation a necessary requirement for exercise to exert its neuroprotective effects, or merely an incidental accompanying phenomenon [19]? To establish a clear causal relationship, the most direct approach is to confirm whether exercise’s beneficial effects are abrogated when the Nrf2 pathway is selectively inhibited.

To address this key question, we used ML385, a highly specific small-molecule Nrf2 inhibitor that suppresses Nrf2 transcriptional activity by blocking its interaction with the CREB-binding protein (CBP). Using a chronic MPTP/probenecid mouse model that accurately recapitulates the progressive pathological course of human Parkinson’s disease (PD), we combined eight weeks of structured treadmill exercise with pharmacological intervention using ML385. We hypothesized that treadmill exercise protects the substantia nigra–striatal dopaminergic system by activating the Nrf2-ARE axis, and that this protective effect can be completely reversed by Nrf2 inhibition. Through multidimensional analyses covering behavioral, histopathological, neurochemical, and molecular biological assessments, this study provides the first direct pharmacological evidence that the Nrf2-ARE pathway is an indispensable mediator of exercise-induced neuroprotection, laying a solid mechanistic foundation for the development of Nrf2-targeted DMT strategies for PD.

## 2. Results

### 2.1. Nrf2 Is Involved in the Improvement of Motor Functions Following Treadmill Exercise

**Figure 1:**
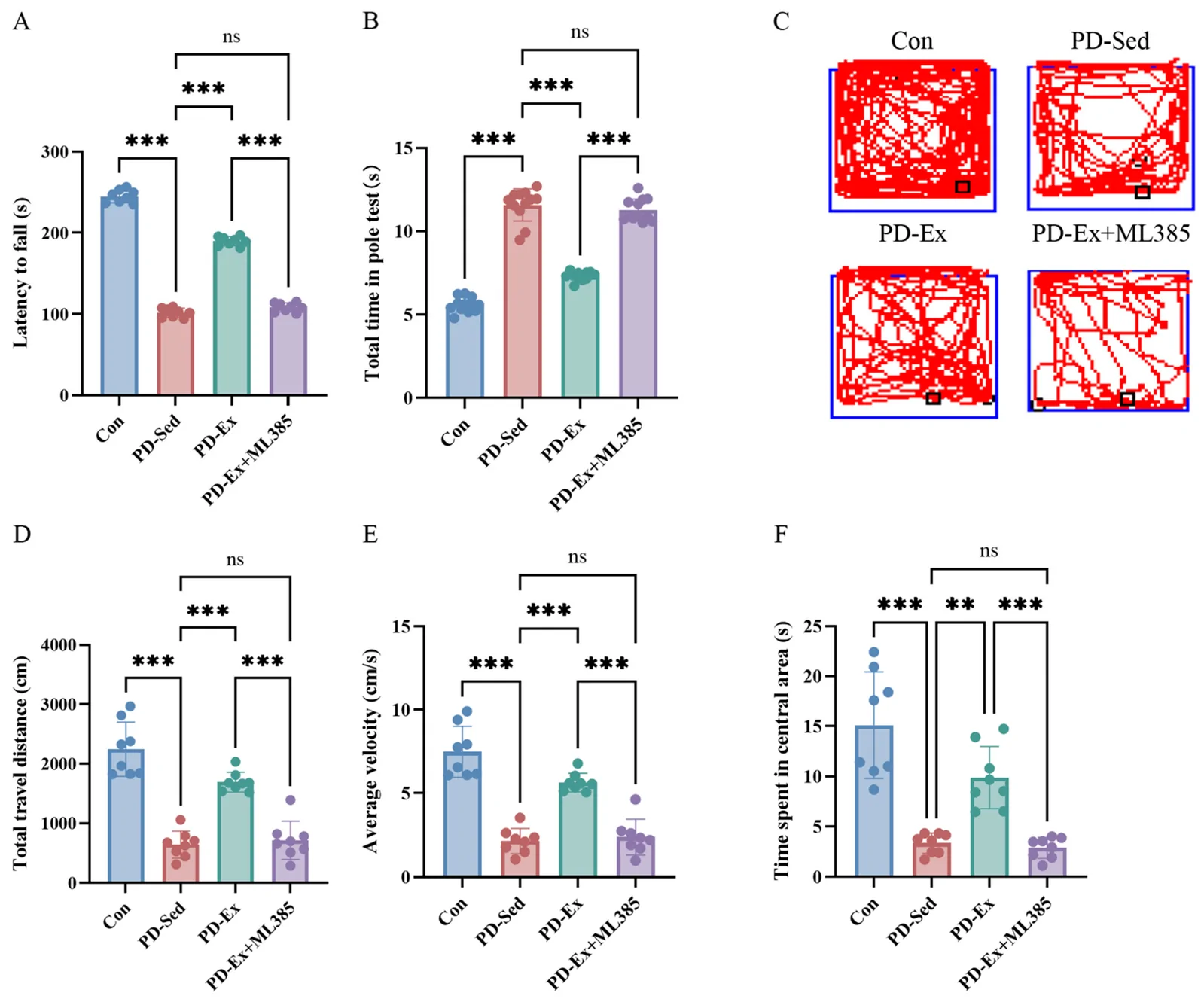
Effects of treadmill exercise on motor coordination in MPTP/p mice and the mediating role of Nrf2. (**A**) Average latency in the rod-crossing test (s). (**B**) Average descent time in the rod-climbing test (s). (**C**) Representative trajectory plots for each group of mice in the open-field test. (**D**) Total distance traveled in the open-field test over 5 min (mm). (**E**) Average speed in the open-field test over 5 min (mm/s). (**F**) Time spent in the central area during the 5 min open-field test (s). Data are expressed as Mean ± SEM, *n* = 8 per group. ** *p* < 0.01, *** *p* < 0.001; ns *p* > 0.05.

Compared to the Control (Con) group (244.92 ± 2.78 s), the rod-tossing latency in the PD-Sed group significantly decreased to 101.75 ± 2.03 s, demonstrating that chronic MPTP/p modeling successfully induced severe motor coordination impairment (Figure 1A). Eight weeks of gradient running treadmill exercise intervention significantly corrected this motor deficit, with the rod-tossing latency in the PD-Ex group rebounding to 189.87 ± 2.01 s (Figure 1A). The results of the rod-climbing experiment show a consistent intergroup trend: mice in the PD-Sed group required 11.58 ± 0.28 s to complete the climb, which is significantly longer than that of the Con group (5.59 ± 0.13 s) (Figure 1B). After exercise intervention, the climbing time in the PD-Ex group shortened to 7.32 ± 0.07 s (Figure 1B); however, this improvement completely disappeared after ML385 treatment, with the climbing time in the PD-Ex+ML385 group rebounding to 11.27 ± 0.19 s (Figure 1B).

The representative activity trajectories of mice in the open-field experiment are shown in Figure 1C. Quantitative analysis revealed that, compared to the Con group, the PD-Sed group exhibited a significant reduction in the 5 min total movement distance (Figure 1D), average movement velocity (Figure 1E), and dwell time in the central area (Figure 1F). Following 8 weeks of treadmill exercise intervention, the PD-Ex group showed a significant increase in the total spontaneous activity distance (Figure 1D), average velocity (Figure 1E), and central area dwell time (Figure 1F) compared to the PD-Sed group. However, when the Nrf2 pathway was inhibited by ML385, all three parameters in the PD-Ex+ML385 group—total movement distance (Figure 1D), average velocity (Figure 1E), and central area dwell time (Figure 1F)—returned to levels comparable to those in the PD-Sed group.

Comprehensive analysis of all behavioral quantification data in Figure 1 confirms that 8 weeks of structured running treadmill exercise effectively ameliorates motor function deficits in chronic PD model mice, and this protective effect is entirely dependent on a fully functional Nrf2 signaling pathway.

### 2.2. Nrf2 Blockade Abolished the Protective Effect of Exercise on Substantia Nigra–Striatal Dopaminergic Function

**Figure 2:**
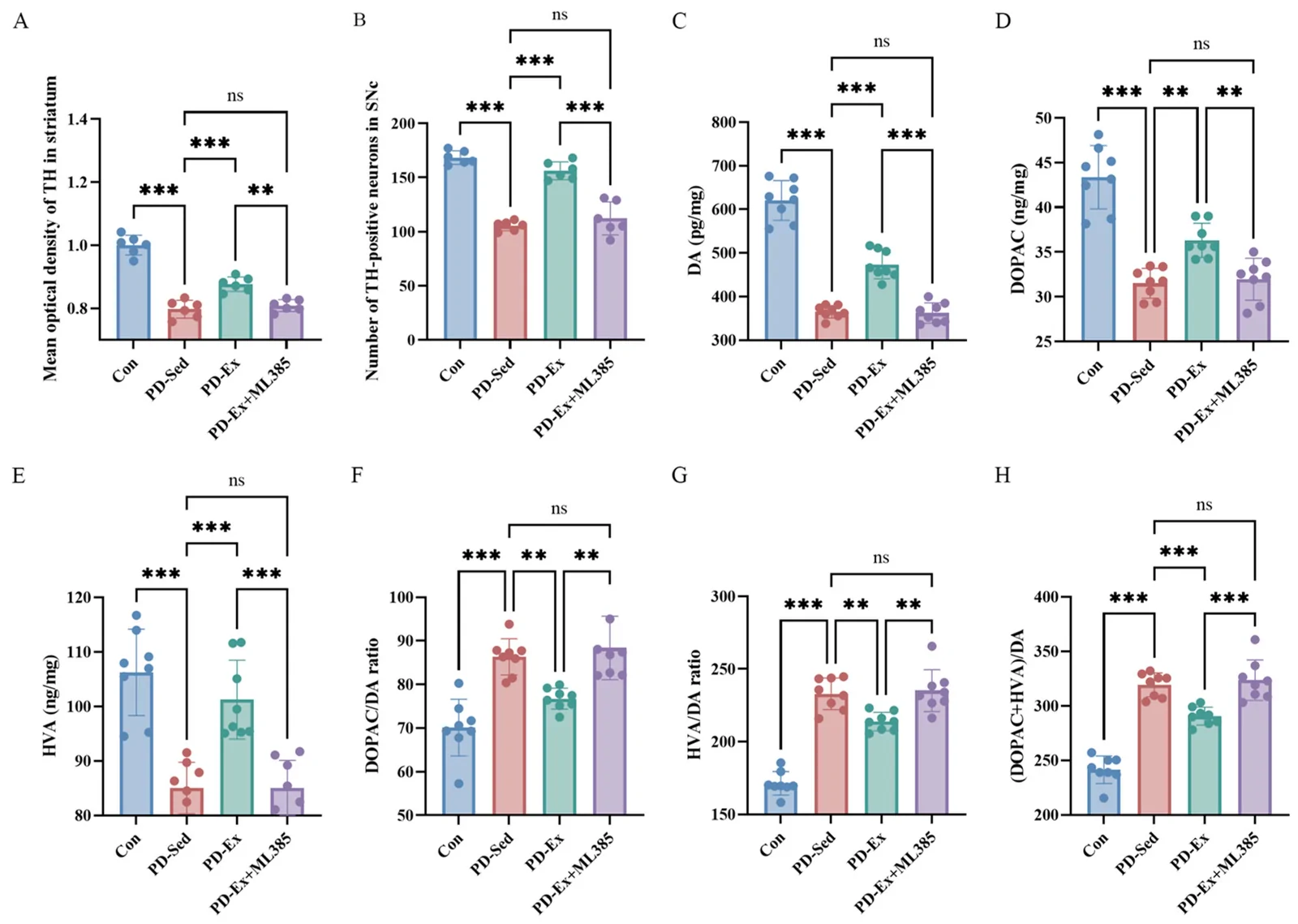
The protective effect of treadmill exercise on the substantia nigra–striatal TH^+^ dopaminergic system and DA function depends on Nrf2. (**A**) Average optical density of striatal TH. (**B**) Number of TH^+^ neurons in the SNc region. (**C**) DA content in the striatum (pg/mg protein). (**D**) DOPAC content in the striatum (ng/mg protein). (**E**) HVA content in the striatum (ng/mg protein). (**F**) DA turnover rate in the striatum: DOPAC/DA ratio. (**G**) DA turnover rate in the striatum: HVA/DA ratio. (**H**) DA turnover rate in the striatum: (DOPAC + HVA)/DA ratio. Data are expressed as mean ± SEM, *n* = 8 per group. ** *p* < 0.01, *** *p* < 0.001; ns *p* > 0.05.

To elucidate the histological basis for exercise-induced improvement of motor function in mice, this study used tyrosine hydroxylase (TH) immunohistochemical staining to examine the morphology of dopaminergic neurons and fibers (Figure S1). Gross microscopic observation showed that in the control (Con) group, TH-positive dopaminergic neurons in the SNc region were densely arranged, with plump and intact cell bodies. In contrast, the PD-sedentary (PD-Sed) group exhibited significant neuronal loss, accompanied by cell body atrophy and deformation (Figure 2B). Quantitative analysis showed that the density of TH-positive neurons in the SNc region of the PD-Sed group was only 105.33 ± 1.84 cells/mm^2^ (Figure 2B). Eight weeks of treadmill exercise significantly alleviated neuronal damage, increasing neuronal density to 156.17 ± 3.38 cells/mm^2^ in the PD-exercise (PD-Ex) group (Figure 2B). However, after co-administration of ML385 to inhibit the Nrf2 pathway, neuronal density in the PD-Ex+ML385 group decreased to 112.17 ± 6.23 cells/mm^2^, with no statistically significant difference compared with the PD-Sed group (Figure 2B).

The intergroup variation pattern of striatal TH fiber optical density was fully consistent with the trend of neuronal counts in the SNc: exercise intervention significantly increased striatal dopaminergic fiber density, while ML385 treatment completely reversed the structural protective effect induced by exercise (Figure 2A). Comprehensive analysis shows that exercise-mediated maintenance of structural integrity in the nigrostriatal dopaminergic neural circuit depends entirely on activation of the Nrf2 pathway.

### 2.3. The Effect of Exercise on Restoring Dopamine Homeostasis in the Striatum Depends on the Activation of the Nrf2 Pathway

ELISA results revealed that the striatal DA level in the Con group was 620.72 ± 16.09 pg/mg protein, while that in the PD-Sed group decreased significantly to 365.14 ± 5.09 pg/mg protein, representing an approximately 41.17% reduction compared with the control group (Figure 2C). Eight weeks of treadmill exercise intervention restored DA levels significantly to 473.72 ± 11.55 pg/mg protein, corresponding to an approximately 42.48% recovery rate relative to the PD-Sed group (Figure 2C). When the Nrf2 pathway was inhibited by ML385, the DA level in the PD-Ex+ML385 group decreased to 362.41 ± 8.23 pg/mg protein, showing no statistically significant difference from the PD-Sed group (Figure 2C).

The quantitative results of striatal DOPAC content showed intergroup trends consistent with those of DA (Figure 2D). The DOPAC content in the PD-Sed group was 31.51 ± 0.59 ng/mg protein, approximately 27.35% lower than that in the Con group (43.37 ± 1.25 ng/mg protein) (Figure 2D); exercise intervention increased DOPAC levels to 36.31 ± 0.67 ng/mg protein, representing an approximately 40.47% recovery compared with the model group (Figure 2D). After ML385 blocked the Nrf2 pathway, DOPAC content decreased again to 31.96 ± 0.83 ng/mg protein, returning to the levels observed in the sedentary model group (Figure 2D).

Statistical analysis of striatal HVA revealed patterns consistent with those observed for DA and DOPAC (Figure 2E). HVA content in the PD-Sed group was 85.03 ± 1.67 ng/mg protein, approximately 19.97% lower than the level of 106.25 ± 2.80 ng/mg protein in the Con group (Figure 2E); treadmill exercise elevated HVA levels to 101.25 ± 2.56 ng/mg protein, representing an approximately 76.44% recovery relative to the model group; in contrast, the PD-Ex+ML385 group exhibited HVA levels of 85.06 ± 1.78 ng/mg protein, which reverted to baseline levels comparable to those observed in the model group (Figure 2E).

The dopamine metabolic turnover ratio indicators DOPAC/DA, HVA/DA, and (DOPAC + HVA)/DA are presented in Figure 2F, Figure 2G, and Figure 2H, respectively. In the control (Con) group, the three turnover ratios were measured as 70.10 ± 2.30 (Figure 2F), 171.31 ± 2.85 (Figure 2G), and 241.41 ± 4.46 (Figure 2H), respectively. In the PD-Sed group, the aforementioned ratios significantly increased via a compensatory mechanism, reaching 86.34 ± 1.46 (Figure 2F), 232.94 ± 3.86 (Figure 2G), and 319.28 ± 3.87 (Figure 2H), which demonstrates that PD model mice develop severe dopamine metabolic disorders in the striatum. Following treadmill exercise intervention, the corresponding ratios in the PD-Ex group decreased to 76.75 ± 0.85 (Figure 2F), 213.79 ± 2.26 (Figure 2G), and 290.53 ± 2.89 (Figure 2H), indicating that exercise effectively corrected the abnormally elevated metabolic values and restored DA turnover to a level close to normal physiological conditions. Nevertheless, after co-administration of ML385 to inhibit Nrf2 activity, the metabolic ratios in the PD-Ex+ML385 group returned to pathological elevated levels, which were 88.39 ± 2.57 (Figure 2F), 235.13 ± 5.12 (Figure 2G), and 323.52 ± 6.55 (Figure 2H), respectively.

Comprehensive analysis of all neurochemical quantification results shown in Figure 2C–H demonstrates that treadmill exercise exerts a protective effect against the impaired synthesis, release and metabolic homeostasis of striatal dopamine neurotransmitters in chronic Parkinson’s disease mice, and this effect is strictly dependent on the activation of the Nrf2 signaling pathway.

### 2.4. Cross-Country Running Exerts Downstream Antioxidant and Anti-Inflammatory Effects by Activating the Nrf2-ARE Pathway

To elucidate the molecular mechanisms by which exercise protects dopaminergic neurons, this study employed nuclear–cytoplasmic separation Western blotting to detect Nrf2 nuclear translocation and downstream HO-1 protein expression, and utilized TH/DHE/NeuN/Iba1 quadruple immunofluorescence staining to quantify mitochondrial ROS levels and activated microglia.

**Figure 3:**
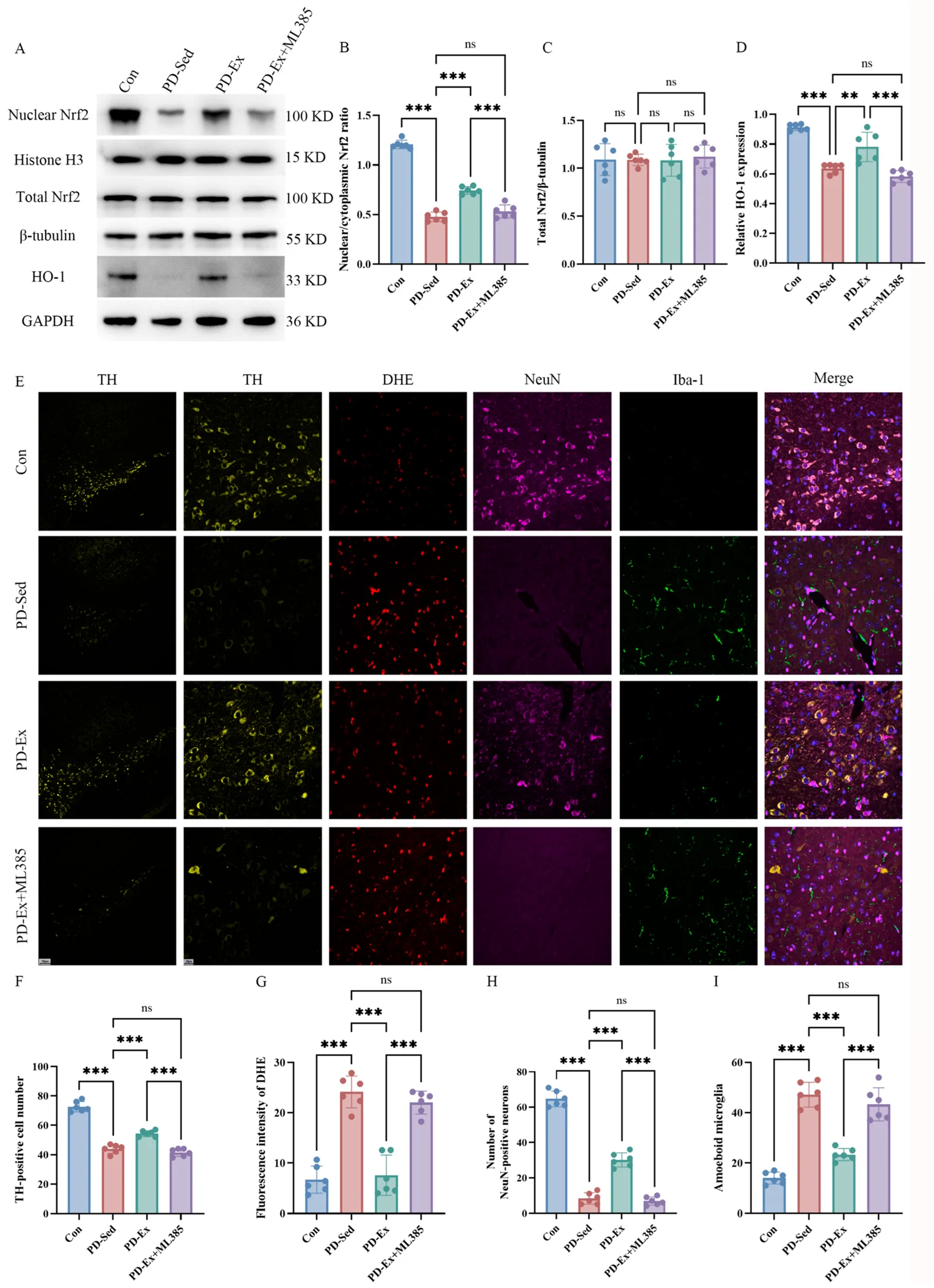
Running on the treadmill exercise inhibits oxidative stress and pro-inflammatory activation of microglia via the Nrf2 pathway. (**A**) Representative Western blot bands. (**B**) Quantitative analysis of the nuclear/cytoplasmic ratio of Nrf2. (**C**) Total Nrf2/β-tubulin. (**D**) Relative expression levels of HO-1 protein. (**E**) Representative images of fluorescence staining for TH, DHE, NeuN, and IBA-1 (scale: 20 μm). (**F**) Number of TH-positive cells. (**G**) Quantitative assessment of DHE fluorescence intensity (reflecting mitochondrial ROS). (**H**) Number of NeuN-positive cells. (**I**) Number of Iba1^+^ ameboid pro-inflammatory microglia (immunofluorescence scale: 50 μm). Data are expressed as mean ± SEM, *n* = 6 per group. ** *p* < 0.01, *** *p* < 0.001; ns *p* > 0.05.

Western blot quantification revealed that the Nrf2 nuclear/cytoplasmic ratio in the PD-Sed group was 0.4783 ± 0.0193, significantly lower than that in the Con group (1.2077 ± 0.0178), demonstrating substantial inhibition of Nrf2 nuclear translocation under pathological PD conditions (Figure 3B). Eight weeks of treadmill exercise intervention significantly reversed this inhibitory effect, with the Nrf2 nuclear/cytoplasmic ratio in the PD-Ex group rising to 0.7446 ± 0.0158 (Figure 3B). After ML385 administration inhibited Nrf2 transcriptional activity, the Nrf2 nuclear/cytoplasmic ratio in the PD-Ex+ML385 group decreased to 0.5315 ± 0.0271, approaching levels observed in the PD-Sed group (Figure 3B). The expression trends of the downstream protective protein HO-1 were completely synchronized with the nuclear translocation efficiency of Nrf2 (Figure 3D). HO-1 expression was downregulated in the PD-Sed group, rebounded after exercise intervention, and was further suppressed following ML385 treatment (Figure 3D).

Quantitative analyses of the immunofluorescence co-localization data revealed the structural and cellular protective effects of exercise. As shown in the cell count data, the number of TH-positive surviving neurons in the substantia nigra was significantly reduced in the PD-Sed group, indicating severe dopaminergic neurodegeneration (Figure 3F). Eight weeks of treadmill exercise markedly alleviated this neuronal loss and preserved the number of TH-positive neurons in the PD-Ex group (Figure 3F). However, this structural protective effect was completely abolished when the Nrf2 pathway was inhibited, with the number of surviving neurons in the PD-Ex+ML385 group declining back to levels comparable to the sedentary model group (Figure 3F). A consistent trend was observed in the quantification of NeuN-positive mature neurons, where exercise effectively mitigated the pathological loss of mature neurons, an effect that was similarly reversed by ML385 intervention (Figure 3H).

At the molecular and inflammatory levels, DHE fluorescence quantification demonstrated a significant accumulation of mitochondrial reactive oxygen species (mtROS) in the substantia nigra of the PD-Sed group (Figure 3G). Treadmill exercise markedly reduced these mtROS levels, demonstrating a robust antioxidant effect; however, this reduction was completely abrogated upon inhibition of the Nrf2 pathway by ML385, causing mtROS levels to rebound (Figure 3G). Furthermore, microglia count data indicated a substantial increase in pro-inflammatory ameboid microglia in the substantia nigra of the PD-Sed group (Figure 3I). Exercise intervention effectively inhibited the activation of microglia toward this pro-inflammatory ameboid phenotype, but this anti-neuroinflammatory benefit was fully reversed by ML385 treatment, resulting in a resurgence of activated ameboid microglia counts (Figure 3I).

Combined analysis of all Western blot (WB) and immunofluorescence quantification data from Figure 3 directly confirms that treadmill exercise exerts its neuroprotective effects primarily through activating the Nrf2-ARE signaling pathway, and this effect is manifested at three levels: molecular activation, oxidative stress regulation, and neuroinflammation modulation.

### 2.5. Weight and General Condition Monitoring in Each Mouse Group

**Figure 4:**
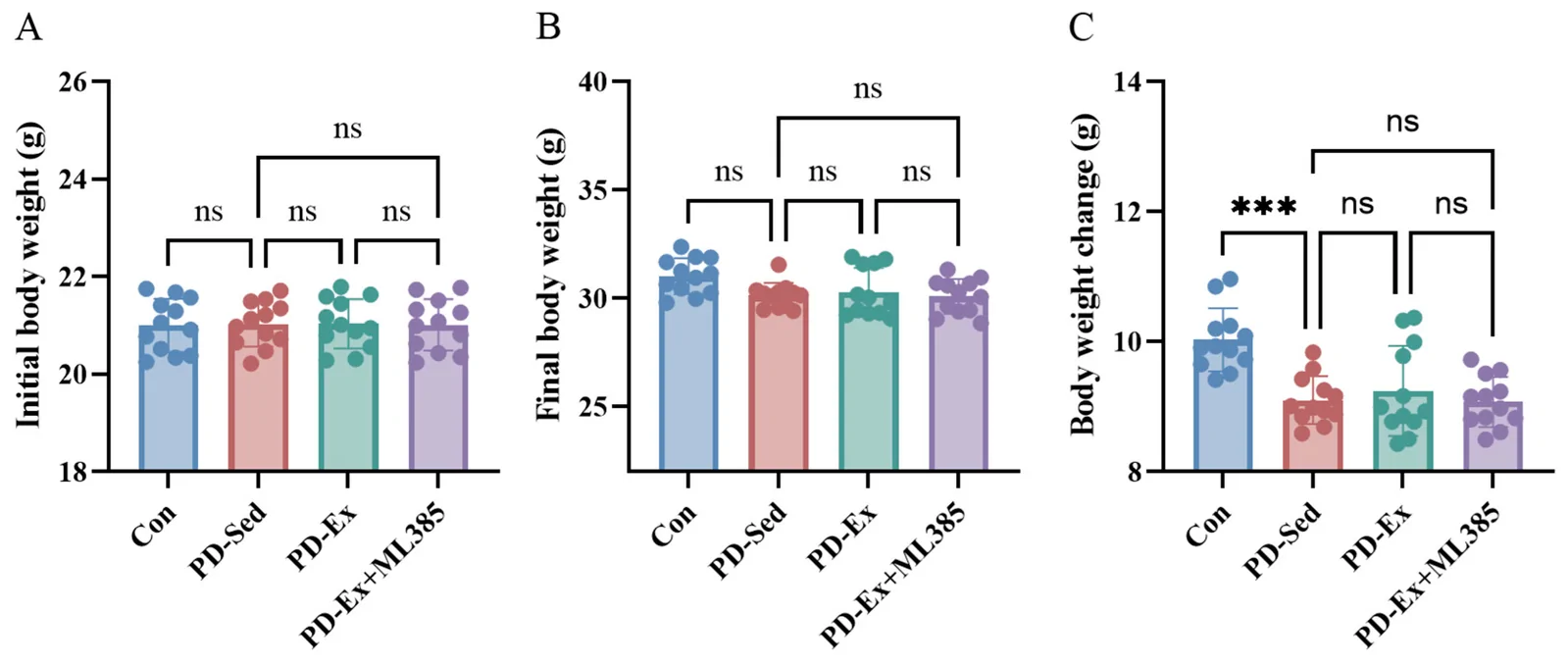
General conditions of animals. (**A**) Initial body weight (g). (**B**) Final body weight (g). (**C**) Change in body weight (g). Data are expressed as mean ± SEM, *n* = 12 per group. *** *p* < 0.001; ns *p* > 0.05.

As illustrated in Figure 4A, no statistically significant intergroup differences in baseline initial body weight were observed among the four experimental groups. From the initiation of the MPTP/p modeling phase, results presented in Figure 4B,C revealed that the rate of body weight gain in the PD-Sed group was significantly lower than that in the Con group, and this significant difference persisted until the termination of the experiment. This finding indicates that PD-induced pathological damage impairs normal metabolic processes and body weight growth in mice.

By comparing body weight measurements across all time points in Figure 4 and Figure S2, no statistically significant differences in body weight were detected between the PD-Ex group and the PD-Sed group, nor between the PD-Ex+ML385 group and the PD-Ex group at any time point during either the modeling or exercise intervention periods. Consistently, no significant differences in final body weight or total body weight change were observed between the PD-Ex+ML385 group and the PD-Sed group.

Collectively, the body weight data obtained from Figure 4 and Figure S2 demonstrate that neither the 8-week moderate-intensity treadmill exercise protocol nor the alternate-day ML385 administration regimen adopted in this study caused additional adverse effects on the body weight of experimental mice, indicating that these two intervention strategies exhibit favorable biosafety and good tolerability in the test animals.

## 3. Discussion

The development of disease-modifying therapies (DMTs) for Parkinson’s disease (PD) remains a significant challenge in the field of neurodegenerative disorders [20,21]. Although exercise has demonstrated clear benefits as a non-pharmacological intervention for improving PD-related motor function, the core molecular mechanisms underlying its neuroprotective effects have not yet been precisely elucidated [22]. Using a chronic MPTP/p mouse model combined with the highly specific Nrf2 inhibitor ML385, this study, for the first time, employed a rigorous in vivo pharmacological blockade strategy to demonstrate that the protective effects of treadmill exercise on the substantia nigra–striatal dopaminergic system are absolutely dependent on activation of the Nrf2-ARE pathway. This finding not only fills a gap in causal evidence regarding the mechanisms of exercise therapy but also provides robust preclinical theoretical support for comprehensive PD rehabilitation strategies targeting Nrf2.

Nrf2 is a prerequisite for exercise-induced neuroprotection. Previous studies primarily remained at the level of phenomenological observation, confirming upregulated Nrf2 expression after exercise but failing to rule out that this is merely an accompanying effect of exercise. The decisive evidence for establishing Nrf2 as the core mediator lies in determining whether the protective effects of exercise disappear when Nrf2 is inhibited. In this study, the application of ML385 provided such conclusive evidence. We found that, without Nrf2 inhibition, 8 weeks of treadmill exercise significantly promoted the nuclear translocation of Nrf2 and expression of downstream HO-1; however, when this pathway was blocked with ML385, improvements in motor coordination and spontaneous activity in PD mice were completely reversed, and behavioral performance returned to the levels observed in the sedentary group. This clear “on-off” effect, in which Nrf2-mediated protection only occurs when Nrf2 is active, robustly rules out alternative compensatory mechanisms and confirms that Nrf2 activation is a necessary prerequisite for exercise-induced neuroprotective effects.

The Nrf2-mediated dual-axis interaction of antioxidation and anti-inflammation reshapes homeostasis of the substantia nigra microenvironment [23,24]. Oxidative stress and neuroinflammation are two interwoven core vicious cycles that drive neuronal death in the pathological network of Parkinson’s disease (PD) [23,25]. Mechanistic data from this study highlight the pivotal role of the Nrf2 pathway in breaking this vicious cycle. Under PD pathology, mitochondrial reactive oxygen species (mtROS) accumulate extensively in the substantia nigra, accompanied by abnormal activation of pro-inflammatory amoeboid microglia. By activating Nrf2, exercise not only suppresses the generation of mtROS at its source but also simultaneously inhibits the transformation of microglia into a pro-inflammatory phenotype. Notably, when ML385 blocks Nrf2, both the antioxidant and anti-inflammatory effects of exercise are completely abolished. This demonstrates that exercise-induced remodeling of microenvironmental homeostasis is not a disordered accumulation of effects across multiple targets, but is highly regulated under the central control of Nrf2 as a transcriptional hub. Nrf2 acts both as an activator for antioxidant genes and as a negative regulatory switch for neuroinflammation.

Improvements to the microenvironment should ultimately manifest as the preservation of dopaminergic neurons and the restoration of their function. This study establishes a complete evidence chain ranging from molecular pathways to synaptic neurotransmitters: specifically, exercise significantly improves the survival rate of TH-positive neurons in the substantia nigra pars compacta and increases striatal fiber density precisely through Nrf2-mediated microenvironmental remodeling; at the neurochemical level, exercise not only substantially elevates dopamine levels in the striatum but also normalizes abnormally elevated dopamine turnover rates, indicating that homeostasis of presynaptic neurotransmitter release and metabolism has been restored. However, when Nrf2 is inhibited, all these protective effects—from neuronal survival to neurotransmitter release—are completely abolished. This multidimensional, full-chain reversal phenomenon further underscores the irreplaceable role of Nrf2 in exercise-induced neuroprotection.

Research limitations and future prospects. Although our study establishes treadmill exercise as an effective Nrf2-dependent neuroprotective intervention for chronic PD, maintaining long-term exercise adherence in patients with advanced motor symptoms remains a clinical challenge. Therefore, pharmacological activation of the Nrf2-ARE pathway represents a highly promising disease-modifying strategy. Several specific Nrf2 activators with distinct mechanisms have shown preclinical or clinical potential. Dimethyl fumarate (DMF), an FDA-approved drug for multiple sclerosis, covalently modifies Keap1 cysteine residues to liberate Nrf2, and has demonstrated significant neuroprotection in PD models. Bardoxolone methyl (CDDO-Me), a semi-synthetic triterpenoid, is a potent inducer of the Nrf2 pathway currently undergoing clinical trials for chronic kidney disease, and has shown efficacy in mitigating neuroinflammation and oxidative stress in neurodegenerative paradigms. Additionally, synthetic oleanane triterpenoids (e.g., RTA 408) and natural sulforaphane have also been identified as effective Nrf2 activators with favorable safety profiles. Given that our findings identify Nrf2 as an indispensable mechanistic node linking exercise to neuroprotection, we propose that these pharmacological Nrf2 activators could be utilized as “exercise mimetics” to replicate the neuroprotective benefits for patients unable to engage in rigorous physical activity. Furthermore, combined interventions pairing moderate exercise with sub-therapeutic doses of Nrf2 activators (such as DMF or sulforaphane) may yield synergistic effects, maximizing Nrf2-ARE pathway activation while minimizing drug-related side effects, offering a robust combinatorial strategy for PD rehabilitation.

## 4. Materials and Methods

### 4.1. Experimental Animals and Grouping

A total of 60 SPF-grade male C57BL/6J mice (8 weeks old, weighing 20–22 g) were used in this experiment, purchased from Beijing Huafukang Biotechnology Co., Ltd., Beijing, China. All mice were housed in a standard environment (temperature: 22 ± 1 °C; relative humidity: 55 ± 5%; 12 h light/12 h dark cycle) with free access to food and water. After one week of acclimatization, the mice were randomly divided into four groups using a random number table, with *n* = 15 per group.

Control group (Con): received intraperitoneal injection of 5% DMSO-probenecid solvent prepared in physiological saline, and underwent treadmill sedentary training without ML385 injection.

Model sedentary group (PD-Sed): underwent chronic MPTP/p modeling, was subsequently kept in a sedentary treadmill environment with no treadmill exercise, and received an equal volume of solvent injection.

Model treadmill group (PD-Ex): underwent chronic MPTP/p modeling, received 8 weeks of treadmill exercise, and was injected with an equal volume of solvent.

Model treadmill + Nrf2 inhibitor group (PD-Ex+ML385): underwent chronic MPTP/p modeling, received 8 weeks of treadmill exercise, and was given concurrent intraperitoneal injection of ML385.

**Figure 5:**
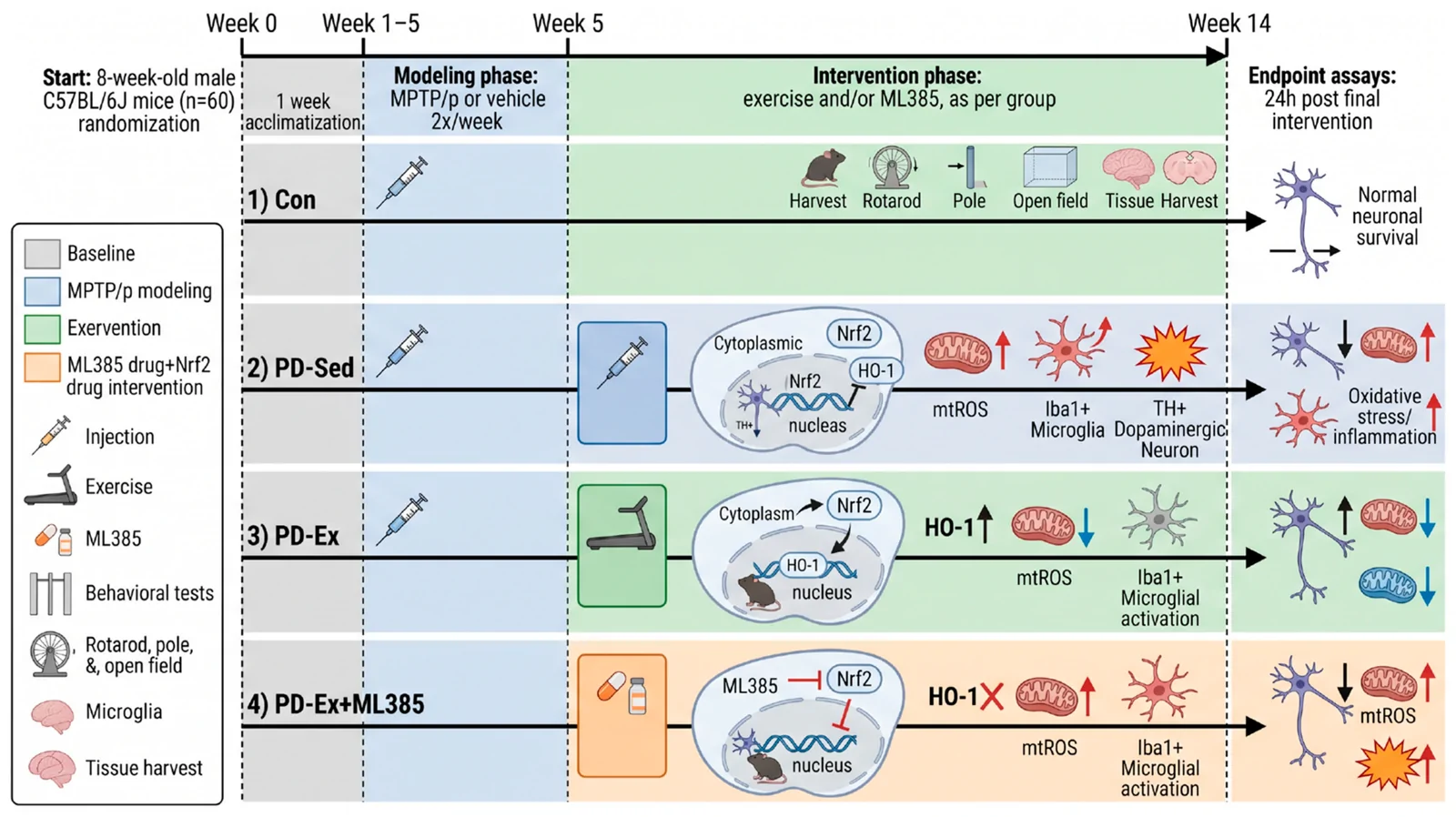
Animal experiment protocol. Note: This figure demonstrates the timeline for adaptive housing, 5-week chronic MPTP/p model establishment, ML385 pre-administration, 8-week treadmill exercise, and final behavioral and biochemical assays.

All experimental procedures strictly complied with the National Guidelines for the Management and Use of Laboratory Animals and were approved by the Experimental Animal Ethics Committee of Hunan Normal University (Approval No.: 2026-0021). Detailed experimental protocols are shown in Figure 5.

### 4.2. Construction of Chronic PD Models and Pharmacological Interventions

To precisely reproduce the progressive pathological characteristics of human Parkinson’s disease (PD), the present study constructed a chronic PD mouse model via the combined administration of 1-methyl-4-phenyl-1,2,3,6-tetrahydropyridine (MPTP, Sigma-Aldrich, St. Louis, MO, USA, Cat. No. M0896) and probenecid. Specifically, MPTP·HCl (Sigma-Aldrich, St. Louis, MO, USA, Cat. No. M0896) was dissolved in physiological saline supplemented with 5% (*v*/*v*) dimethyl sulfoxide (DMSO, Sigma-Aldrich, St. Louis, MO, USA, Cat. No. 34869), while probenecid (MCE, Shanghai, China, Cat. No. HY-B0545) was dissolved in phosphate-buffered saline (PBS, Serviebio, Wuhan, China, Cat. No. G4202) adjusted to pH 7.4 with 0.1 N NaOH. During model establishment, mice received an intraperitoneal injection of probenecid at a dose of 250 mg/kg, followed by an intraperitoneal injection of MPTP at 25 mg/kg 60 min after probenecid administration. This procedure was performed twice weekly (once every 3.5 days) over a total period of 5 weeks, resulting in 10 administrations. Mice in the control (Con) group received an equal volume of the corresponding solvent at identical time points.

To elucidate the causal contribution of Nrf2 to motor neuron protection in the pathological context of PD, the present study utilized ML385, a highly specific Nrf2 inhibitor that blocks the interaction between Nrf2 and CREB-binding protein (CBP) to suppress the transcriptional activity of Nrf2. ML385 (MCE, Shanghai, China, Cat. No. HY-100523) was dissolved in DMSO to prepare a 50 mg/mL stock solution, which was stored at −20 °C under light protection. The stock solution was diluted to a working concentration of 2 mg/mL with PBS containing 5% DMSO prior to administration. Based on preliminary experimental results and the previously reported literature, mice in the PD-Ex+ML385 group received intraperitoneal injections of ML385 at a dose of 30 mg/kg, starting one week before the formal exercise intervention. ML385 was administered every other day (three times weekly) throughout the entire 8-week exercise intervention period, resulting in a total administration duration of approximately 9 weeks. Mice in the PD-Sed group and PD-Ex group received an equal volume of solvent (PBS containing 5% DMSO) at the same time points. The Con group received the vehicle for both the MPTP/probenecid solvent and the ML385 solvent, and was maintained under sedentary conditions with treadmill exposure matching other groups.

It should be noted that the present study specifically focused on the disease-modifying effects of exercise and the requirement of Nrf2 activation under PD pathological conditions. Accordingly, non-PD control groups receiving exercise intervention alone or ML385 intervention alone (i.e., Con-Ex and Con-Ex+ML385) were not included in the current experimental design. Although the inclusion of these groups would help explicitly exclude non-specific pharmacological and physiological effects of ML385 and exercise in healthy animals, the primary objective of this study was to test whether the neuroprotective effect of exercise on the MPTP-lesioned dopaminergic system is eliminated after Nrf2 inhibition. Future studies incorporating healthy exercise and Nrf2 inhibition cohorts are required to fully clarify the baseline and pleiotropic effects of Nrf2 modulation.

### 4.3. Running Platform Exercise Intervention

Exercise intervention was implemented utilizing a mouse treadmill system (Guangzhou Feidi Biotechnology Co., Ltd., Model FD000043, Guangzhou, China), with all training sessions conducted between 9:00 AM and 12:00 PM to control for circadian confounding effects. The training protocol was developed in accordance with well-validated moderate-intensity aerobic exercise paradigms for Parkinson’s disease rodent models, which are optimized to induce neuroprotective effects without triggering exhaustive physiological stress. Before the commencement of formal intervention, mice assigned to exercise intervention groups (PD-Ex and PD-Ex+ML385) completed a 5-day adaptation period, during which animals ran at 5 m/min for 15 min daily to acclimate to the treadmill apparatus and establish consistent running patterns. The formal intervention procedure spanned 8 consecutive weeks, with training arranged from Monday to Friday and rest permitted during weekends. To facilitate progressive physiological adaptation, exercise intensity was gradually elevated over the intervention period: weeks 1–2 (running speed: 8 m/min, 30 min per day); weeks 3–4 (running speed: 10 m/min, 30 min per day); weeks 5–8 (running speed: 10 m/min, 40 min per day). The employed running speeds meet the criteria for moderate aerobic exercise in mice, generally remaining below the anaerobic threshold and reducing inter-individual variability in exercise-induced exhaustion. For control of environmental confounders, animals in the blank control group and sedentary Parkinson’s disease model group were placed on the powered-off stationary treadmill for equivalent durations during each training session to eliminate potential differences in environmental stress exposure.

### 4.4. Behavioral Testing

Twenty-four hours after the completion of the final exercise intervention, behavioral tests were conducted in a quiet, dimly lit room by an investigator unaware of the group assignments.

Rotarod Test: Evaluates motor coordination and learning ability. Mice are placed on a rotating rod apparatus, (with rotational speed linearly increased from 4 rpm to 35 rpm, and the time from initiation of rotation until the mouse falls off the rod (latency period) is recorded, with an upper limit of 300 s. Each mouse undergoes five tests spaced 30 min apart, and the results are averaged.

Pole Test: Evaluates motor initiation and posture control abilities. The mouse is placed head-up at the top of a vertical wooden pole (50 cm long, 1.5 cm in diameter), and the time required for it to turn around and crawl to the bottom is recorded, with a maximum duration of 60 s.

Open Field Test: Evaluates spontaneous activity and anxiety-like behaviors. Mice are placed in a 50 × 50 × 40 cm open box, and the total distance traveled, average speed, and time spent in the central area are recorded over a 5 min period.

### 4.5. Sample Collection and Immunohistochemical (IHC) Staining (TH Immunohistochemistry)

Following behavioral testing, mice were deeply anesthetized, and brain tissues were perfused with 4% polyformaldehyde (PFA, Sigma-Aldrich, St. Louis, MO, USA, Cat. No. 158127) for fixation. The brain tissues were subsequently post-fixed, gradient dehydrated, and embedded in paraffin to prepare 8 μm coronal sections containing the substantia nigra pars compacta and striatum. After dewaxing, antigen retrieval, and blocking, rabbit anti-TH monoclonal primary antibody (1:500, Abcam, Cambridge, UK, Cat. No. ab137721) was added and incubated overnight at 4 °C, followed by washing, addition of HRP-labeled secondary antibody, DAB staining, and counterstaining with hematoxylin. Images were acquired under optical microscopy, and the number of TH-positive neurons was counted by two independent blinded researchers. The density of TH-positive fibers in the striatum was quantified using Image-Pro Plus 6.0 software via measuring the mean optical density (MOD).

### 4.6. Neurochemical Analysis of Dopamine and Its Metabolites in the Striatum

Right-side striatal tissue was excised, precisely weighed, and treated with pre-cooled 0.1 M perchloric acid (PCA, Sigma-Aldrich, St. Louis, MO, USA, Cat. No.311413) solution to prepare a 10% tissue homogenate on ice. The homogenate was then centrifuged at 12,000× *g* for 15 min at 4 °C, and the supernatant was filtered through a 0.22 μm membrane. Using a competitive ELISA kit from Jiangsu Adison Biotechnology Co., Ltd., dopamine (DA, AIDISHENG, Yancheng, China, Cat. No. ADS-3579O1), dihydroxyphenylacetic acid (DOPAC, Mediteda Biopharmaceutical Technology, Shanghai, China, Cat. No. YJD-ELISA-PD-011), and homovanillic acid (HVA, Mediteda Biopharmaceutical Technology, Shanghai, China, Cat. No. YJD-ELISA-PD-017) concentrations were measured strictly according to the manufacturer’s protocol. All samples were tested in duplicate wells, with intra-plate and inter-plate coefficients of variation (CV) controlled within 10% and 15%, respectively. Protein concentrations were determined by the BCA method, and the results were expressed as pg/mg protein. The ratios of DOPAC/DA and HVA/DA were calculated to assess dopamine turnover rates.

### 4.7. Protein Blot Analysis of Nrf2 Nuclear Translocation

Tissue samples from the contralateral midbrain were collected, and nuclear and cytoplasmic proteins were extracted using a nuclear protein extraction kit (Abcam, Cambridge, UK, Cat. No. ab113474). After quantification via BCA assay, 30 μg of protein was separated by SDS-PAGE and transferred onto PVDF membranes. The membranes were blocked with 5% skim milk, then incubated overnight at 4 °C with the following primary antibodies: anti-Nrf2 (1:1000, Abcam, Cambridge, UK, Cat. No. ab325240), anti-HO-1 (1:1000, Abcam, Cambridge, UK, Cat. No. Cambridge, UK, Cat. No. ab68477), anti-Histone H3 (nuclear internal standard, 1:5000, Huabio, Hangzhou, China, Cat. No. ET170164), GAPDH (1:20,000, Huabio, Hangzhou, China, Cat. No. HA724237H) and anti-β-tubulin (cytoplasmic internal standard, 1:5000, Huabio, Hangzhou, China, Cat. No. HA724236H). After washing the membranes, HRP-labeled secondary antibodies (1:10,000, Huabio, Hangzhou, China, Cat. No. HA1001) were added, followed by ECL (Mediteda Biopharmaceutical Technology, Shanghai, China, Cat. No. YJD-WB077) chemiluminescent development. Grayscale values were analyzed using ImageJ software (version 1.52a, National Institutes of Health, Bethesda, MD, USA). Nrf2 nuclear translocation efficiency was quantified by calculating the ratio of [Nrf2 (nucleus)/Histone H3] to [Nrf2 (cytoplasm)/β-tubulin].

### 4.8. Immunofluorescence and Evaluation of Oxidative Stress/Nerve Inflammation

Fresh brain tissue was prepared into 8 μm frozen sections. The sections were incubated at 37 °C for 30 min in the dark with 2.5 mM dihydroethidium bromide (DHE, Sigma, Sigma-Aldrich, St. Louis, MO, USA, Cat. D7008) to specifically label mitochondrial reactive oxygen species, then fixed with 4% PFA and blocked with 5% BSA. Subsequently, a mixture of primary antibodies—rabbit anti-TH (1:500, Abcam, Cambridge, UK, Cat. No. ab137721), guinea pig anti-NeuN (1:1000, Huabio, Hangzhou, China, Cat. No. HA601481), and chicken anti-Iba1 (1:500, Huabio, Hangzhou, China, Cat. No. HA601376)—was added, and the sections were incubated overnight at 4 °C. After washing, a mixture of fluorescent secondary antibodies (Alexa Fluor 488, Abcam, Cambridge, UK, Cat. No. ab150073; iFluor 594, Huabio, Hangzhou, China, Cat. HA1143; iFluor 647, Abcam, Cambridge, UK, Cat. No. ab150171. 1:500) and DAPI (1:1000, Sigma-Aldrich, St. Louis, MO, USA, Cat. D9542) was added, followed by incubation at room temperature in the dark for 1 h. Images were acquired using a multicolor fluorescence microscope (Mediteda Biopharmaceutical Technology, Shanghai, China, Cat. No. YJD-NE610). mtROS levels were quantified by measuring the average fluorescence intensity of DHE. Microglial morphology was classified as either ramified resting type or ameboid pro-inflammatory activated type, and statistical analysis was performed by counting the number of ameboid microglia per field of view.

### 4.9. Statistical Analysis

All data were analyzed using GraphPad Prism 8.0 software and expressed as mean ± standard error. Inter-group comparisons were performed using one-way ANOVA. If homogeneity of variances was met, Tukey’s post hoc test was employed; if heterogeneity of variances was present, Welch’s one-way ANOVA was used. A *p*-value < 0.05 was considered statistically significant.

## 5. Conclusions

In conclusion, through rigorous in vivo pharmacological interventions, this study provides the first direct causal evidence that the neuroprotective effects of treadmill exercise in chronic PD model mice are strictly dependent on activation of the Nrf2-ARE pathway. Nrf2 not only serves as a defense mechanism against exercise-induced oxidative stress, but also acts as a central regulatory hub that suppresses neuroinflammation and rescues dopaminergic neurons. This finding advances our understanding of the molecular mechanisms underlying the concept of “exercise is medicine”, and provides solid experimental evidence for developing Nrf2-targeted disease-modifying therapies and comprehensive rehabilitation strategies for PD.

## Data Availability

All data supporting the findings of this study are available from the corresponding author upon request.

## Supplementary Materials

The following supporting information can be downloaded at: https://www.mdpi.com/article/10.3390/ijms27167439/s1.

## References

[1] Pain S., Deguil J., Belin J., Barraud P., Ragot S., Houeto J.-L. (2019). Regulation of Protein Synthesis and Apoptosis in Lymphocytes of Parkinson Patients: The Effect of Dopaminergic Treatment. Neurodegener. Dis..

[2] Liu X., Wei B., Bi Q., Sun Q., Li L., He J., Weng Y., Zhang S., Mao G., Bao Y. (2020). MPTP-Induced Impairment of Cardiovascular Function. Neurotox. Res..

[3] Wen P., Sun Z., Gou F., Wang J., Fan Q., Zhao D., Yang L. (2025). Oxidative stress and mitochondrial impairment: Key drivers in neurodegenerative disorders. Ageing Res. Rev..

[4] Wenbo Z., Zhigang M., Jiren Z., Ce Z., Ting L., Pinyuan Z., Tian W. (2023). Antinociceptive effects of carbidopa levodopa on normal rats and Parkinson’s disease mice. Pak. J. Pharm. Sci..

[5] Chase T.N., Al-Sabbagh A., Koga M., Clarence-Smith K. (2024). Reduced Plasma Levodopa Fluctuations with More Frequent Administration of a Novel Carbidopa/Levodopa Functionally Scored Tablet. Clin. Pharmacol. Drug Dev..

[6] Guo Z., Mo Z. (2020). Keap1-Nrf2 signaling pathway in angiogenesis and vascular diseases. J. Tissue Eng. Regen. Med..

[7] Gureev A.P., Chernyshova E.V., Krutskikh E.P., Sadovnikova I.S., Tekutskaya E.E., Dorohova A.A. (2024). Role of the Nrf2/ARE Pathway in the mtDNA Reparation. Front. Biosci..

[8] Dovinova I., Kvandova M., Balis P., Gresova L., Majzunova M., Horakova L., Chan J., Barancik M. (2020). The Role of Nrf2 and PPARγ in the Improvement of Oxidative Stress in Hypertension and Cardiovascular Diseases. Physiol. Res..

[9] Kopacz A., Kloska D., Forman H.J., Jozkowicz A., Grochot-Przeczek A. (2020). Beyond repression of Nrf2: An update on Keap1. Free Radic. Biol. Med..

[10] Zhang Z., Peng L., Fu Y., Wang W., Wang P., Zhou F. (2021). Ginnalin A Binds to the Subpockets of Keap1 Kelch Domain To Activate the Nrf2-Regulated Antioxidant Defense System in SH-SY5Y Cells. ACS Chem. Neurosci..

[11] Bento-Pereira C., Dinkova-Kostova A.T. (2020). Activation of transcription factor Nrf2 to counteract mitochondrial dysfunction in Parkinson’s disease. Med. Res. Rev..

[12] Salvadè M., Gardoni F. (2025). Drug repurposing of dimethyl fumarate in Parkinson’s disease: A promising disease-modifying strategy. Eur. Rev. Med. Pharmacol. Sci..

[13] Han C., Zhang H., Wang Q., Meng P., Hou Y., Zhang Y. (2025). Physical exercise and inflammation in Parkinson’s disease: A review. Neurol. Sci..

[14] Ernst M., Folkerts A.-K., Gollan R., Lieker E., Caro-Valenzuela J., Adams A., Cryns N., Monsef I., Dresen A., Roheger M. (2024). Physical exercise for people with Parkinson’s disease: A systematic review and network meta-analysis. Cochrane Database Syst. Rev..

[15] Feng Y.-S., Yang S.-D., Tan Z.-X., Wang M.-M., Xing Y., Dong F., Zhang F. (2020). The benefits and mechanisms of exercise training for Parkinson’s disease. Life Sci..

[16] Palasz E., Gasiorowska-Bien A., Drapich P., Niewiadomski W., Niewiadomska G. (2025). Steady Moderate Exercise Confers Resilience Against Neurodegeneration and Neuroinflammation in a Mouse Model of Parkinson’s Disease. Int. J. Mol. Sci..

[17] Palasz E., Wysocka A., Gasiorowska A., Chalimoniuk M., Niewiadomski W., Niewiadomska G. (2020). BDNF as a Promising Therapeutic Agent in Parkinson’s Disease. Int. J. Mol. Sci..

[18] Ostrom E.L., Traustadóttir T. (2020). Aerobic exercise training partially reverses the impairment of Nrf2 activation in older humans. Free. Radic. Biol. Med..

[19] Levings D.C., Pathak S.S., Yang Y.-M., Slattery M. (2023). Limited expression of Nrf2 in neurons across the central nervous system. Redox Biol..

[20] Kalia L.V., Asis A., Arbour N., Bar-Or A., Bove R., Di Luca D.G., Fon E.A., Fox S., Gan-Or Z., Gommerman J.L. (2024). Disease-modifying therapies for Parkinson disease: Lessons from multiple sclerosis. Nat. Rev. Neurol..

[21] Zhang S., Shao G., Wu B., Xia L., Wang L., Li L., Jin K., Zou Y., Sun C. (2025). Progress in Disease-Modifying Therapies for Parkinson’s Disease. Aging Dis..

[22] Mitchell A.K., Bliss R.R., Church F.C. (2024). Exercise, Neuroprotective Exerkines, and Parkinson’s Disease: A Narrative Review. Biomolecules.

[23] Gao Y., Tang X., Yao J., Sun T., Chen Y., Cheng C., Yang J., Wang B., Liu A., Yang L. (2025). Targeting the bile acid receptor TGR5 with Gentiopicroside to activate Nrf2 antioxidant signaling and mitigate Parkinson’s disease in an MPTP mouse model. J. Adv. Res..

[24] Linker R.A., Lee D.-H., Ryan S., van Dam A.M., Conrad R., Bista P., Zeng W., Hronowsky X., Buko A., Chollate S. (2011). Fumaric acid esters exert neuroprotective effects in neuroinflammation via activation of the Nrf2 antioxidant pathway. Brain.

[25] Zhang C., Zhao M., Wang B., Su Z., Guo B., Qin L., Zhang W., Zheng R. (2021). The Nrf2-NLRP3-caspase-1 axis mediates the neuroprotective effects of Celastrol in Parkinson’s disease. Redox Biol..

