# Supplementary material for: Activation of the Nrf2-ARE Signaling Pathway Mediates the Neuroprotective Effects of Exercise in a Mouse Model of Chronic Parkinson’s Disease
Source: Int J Mol Sci. 2026 Aug 20;27(16):7439. doi: 10.3390/ijms27167439 (PMC13513865; doi:10.3390/ijms27167439)
Supplement: Supplementary file 1 [file ijms-27-07439-s001.zip › ijms-4470698-supplementary.pdf]

Supplementary Figure S1

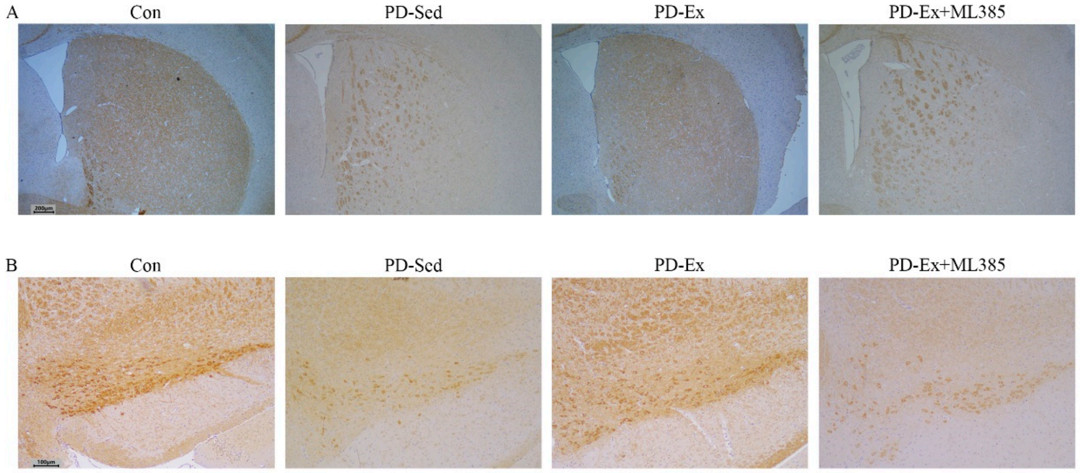

Supplementary Figure S1. (A) Representative immunohistochemical staining image of striatal TH (scale bar: 200 μm); (B) Representative immunohistochemical staining image of TH in the SNc region (scale bar: 100 μm);

Supplementary Figure S2

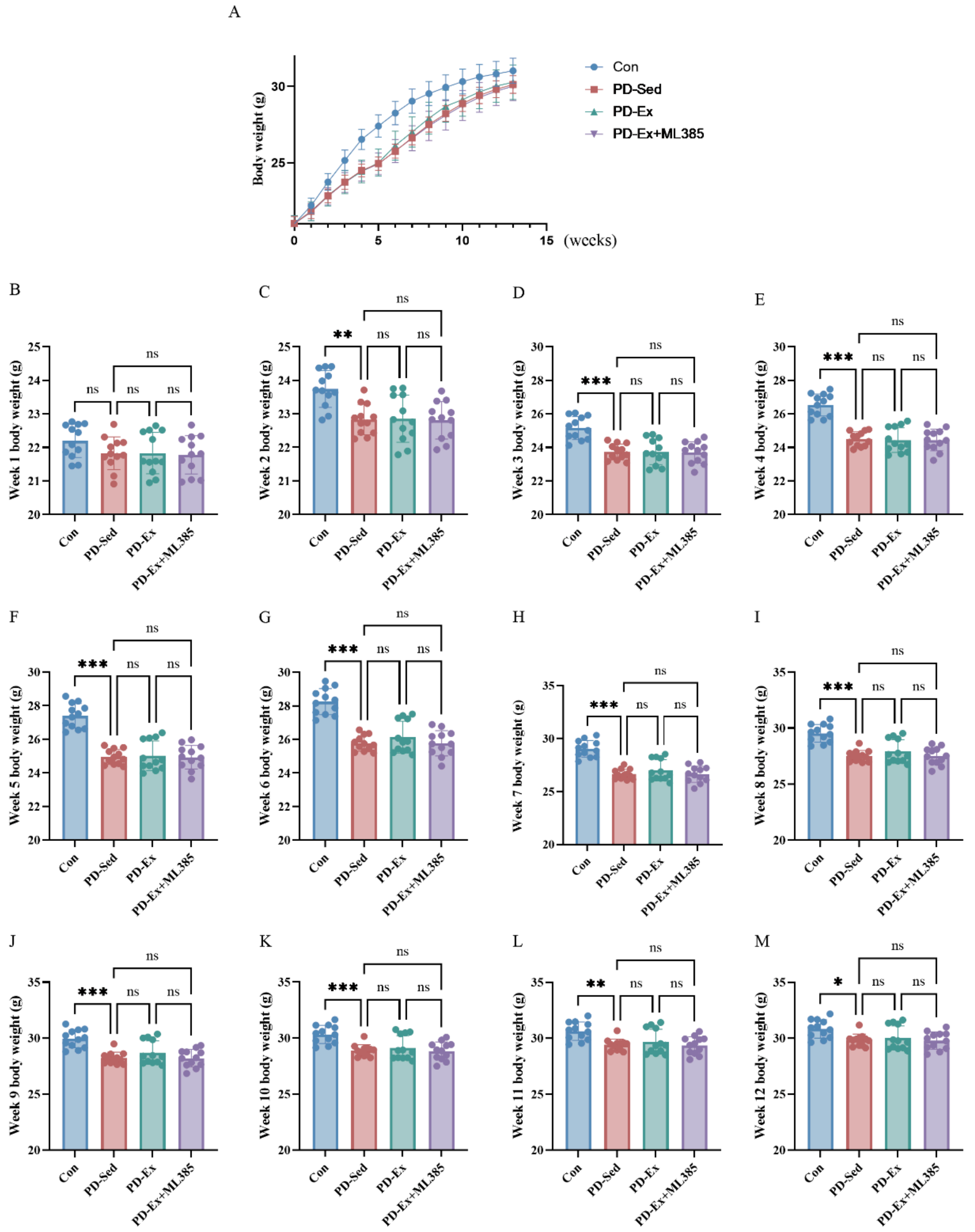

Supplementary Figure S2 comprehensively illustrates the modeling results and weekly average body weights of mice throughout the entire exercise cycle: (A) weekly average weights across all four groups; (B–M) weekly inter-group weight comparisons from weeks 1 to 12, respectively. Repeated-measures ANOVA revealed that only the PD-Sed group exhibited significantly lower overall body weight than the Con group, while exercise intervention and inhibitor administration did not alter the trend in weight changes. Data are presented as Mean ± SEM, n = 12 per group. *P <0.05, **P <0.01, ***P <0.001; ns P> 0.05.

Supplementary Figure S3

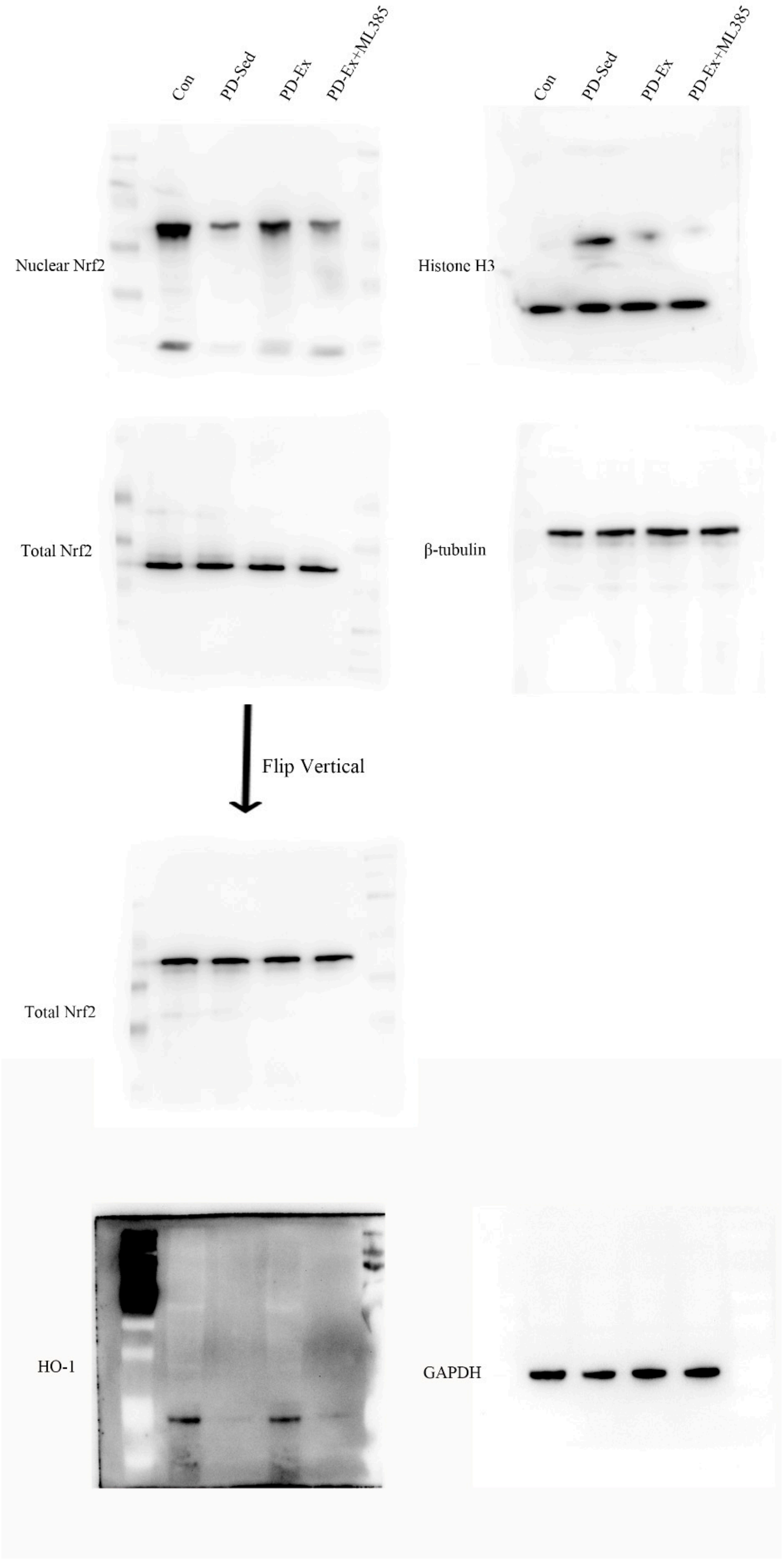

Supplementary Figure S3 Full Western blot membrane images for target proteins and loading controls.
